# Risk factors and clinical characteristics for *Stenotrophomonas maltophilia* infection in an acute care hospital in Japan: a single-center retrospective study

**DOI:** 10.1186/s40780-025-00429-2

**Published:** 2025-03-28

**Authors:** Michiya Tanuma, Takayuki Sakurai, Hidemasa Nakaminami, Masayo Tanaka

**Affiliations:** 1 Department of Pharmacy, NTT Medical Center Tokyo, 5-9-22 Higashi-Gotanda, Shinagawa-Ku, Tokyo, 141-8625 Japan; 2 Department of Clinical Microbiology, School of Pharmacy, Tokyo University of Pharmacy and Life Sciences, 1432-1 Horinouchi, Hachioji, Tokyo 192-0392 Japan; 3 Department of Infectious Diseases, NTT Medical Center Tokyo, 5-9-22 Higashi-Gotanda, Shinagawa-Ku, Tokyo, 141-8625 Japan

**Keywords:** *Stenotrophomonas maltophilia*, Risk factors, Pneumonia, Antimicrobial susceptibility, Antimicrobial resistance

## Abstract

**Background:**

*Stenotrophomonas maltophilia* (*S. maltophilia*) is a Gram-negative pathogen that causes opportunistic infections. Although the mortality rate among patients with nosocomial infections caused by *S. maltophilia* is high, the risk factors for infection vary among studies. Moreover, *S. maltophilia* is highly resistant to several classes of antimicrobial agents. To date, few studies on *S. maltophilia* have been conducted in Japan, and the details remain unclear. Therefore, the objective of this study was to investigate the risk factors associated with *S. maltophilia* infection and the antimicrobial susceptibility of *S. maltophilia* isolates identified in our hospital.

**Methods:**

In this study, we investigated the risk factors associated with *S. maltophilia* infection and clinical characteristics isolated from patients at the NTT Medical Center Tokyo (Tokyo, Japan). We retrospectively examined the *S. maltophilia* isolates and the corresponding patients between March 2022 and August 2023.

**Results:**

Fifty-eight patients with *S. maltophilia* isolated (median age, 80.5 years; age range, 49–100 years; 70.7% male) were enrolled in this study. Twelve cases (20.7%) were placed in the *S. maltophilia* infection group and 46 cases were placed in the *S. maltophilia* colonization group. Central venous (CV) catheterization and higher Sequential Organ Failure Assessment (SOFA) scores were identified as risk factors for *S. maltophilia* infection. In addition, the 30-day mortality rate was significantly higher, and the survival rate was significantly lower in patients with *S. maltophilia* infection. The antimicrobial susceptibility rates of *S. maltophilia* were as follows: 28.6% for ceftazidime, 2.4% for cefozopran, 96.6% for levofloxacin, 100% for minocycline, and 98.3% for trimethoprim-sulfamethoxazole.

**Conclusions:**

In actual clinical practice, *S. maltophilia* was more frequently isolated from sputum. However, most of the cases were colonization, and cases of infection were rare. Early treatment initiation should be considered for *S. maltophilia* infection in cases where the pathogen is detected from sterile sites, such as blood cultures and pleural fluid or from sputum in cases with a high SOFA score and CV catheter insertion.

## Background

*Stenotrophomonas maltophilia* (*S. maltophilia*) is a Gram-negative pathogen that causes a variety of infections, primarily pneumonia and bloodstream infections. In the hospital environment, it is isolated from sources such as tap water, nebulizers, ventilator circuits, and contaminated disinfectants, making it an important opportunistic pathogen causing healthcare-associated infections [1]. Nosocomial infections caused by *S. maltophilia* have a high mortality rate, ranging between 12.5–41% for bacteremia, 40–50% for pneumonia, and 39% for endocarditis [1, 2]. Risk factors for *S. maltophilia* infection, including malignancy, intravenous device placement, chronic respiratory disease, immunodeficiency, prolonged antimicrobial use, and prolonged hospitalization or intensive care unit (ICU) admission, vary among reports [3–7]. In addition, in a real clinical setting, it is difficult to determine whether a patient is eligible for treatment if *S. maltophilia* is isolated in a non-sterile specimen.

Fluoroquinolones, tetracyclines, and trimethoprim-sulfamethoxazole (TMP-SMX) are commonly used to treat *S. maltophilia* infections [8–10]. However, the resistance rates of *S. maltophilia* to these drugs vary based on region [11]. *S. maltophilia* is highly resistant to several antimicrobial agents and is reported to acquire resistance genes and genetic mutations that limit antimicrobial therapy [12, 13]. Recently, increased resistance of *S. maltophilia* to fluoroquinolones and TMP-SMX has been reported in several countries [14–16]. The emergence of fluoroquinolone- and TMP-SMX-resistant strains limits the choice of antimicrobial agents to treat *S. maltophilia* infections, potentially posing a public health concern in the future. Understanding regional antimicrobial susceptibility is important when using antimicrobial agents. However, there are few reports on the resistance of *S. maltophilia* in Japan, and the characteristics remain unknown. Therefore, in this study, we aimed to investigate the antimicrobial susceptibility of *S. maltophilia* isolates in our hospital and the risk factors for *S. maltophilia* infection.

## Methods

### Study design, setting, and patients

This retrospective study was conducted at the NTT Medical Center in Tokyo, Japan, an acute care hospital with 594 inpatient beds. The monthly antimicrobial use in our hospital during the study period is presented in terms of antimicrobial use density (AUD) and days of therapy (DOT). The mean (standard deviation) values for various antimicrobials were as follows: fluoroquinolones, 0.43 (0.19) AUD, 0.47 (0.17) DOT; tetracyclines, 0.21 (0.07) AUD, 0.20 (0.07) DOT; TMP-SMX, 0.03 (0.04) AUD, 0.02 (0.03) DOT; 3rd-generation cephalosporins, 4.10 (0.72) AUD, 4.75 (1.05) DOT; 4th-generation cephalosporins, 1.27 (0.43) AUD, 1.57 (0.47) DOT; and carbapenems, 2.99 (0.61) AUD, 4.43 (0.86) DOT. The definitions of AUD and DOT were as follows: AUD = [total usage / defined daily dose; DDD × total patient days] × 100 and DOT = [total days of used / total patient days] × 100.

*S. maltophilia* isolates and the patients from whom *S. maltophilia* was isolated were retrospectively examined between March 2022 and August 2023. If multiple strains were isolated from the same patient, the first strain detected was included in this study. There were no cases in which *S. maltophilia* was detected in culture samples from different sites in the same patient. This study was approved by the Ethics Committee of NTT Medical Center Tokyo prior to the study (Approval number: 22–66). Owing to the retrospective and non-invasive nature of the study, the requirement for individual informed consent was waived. Disclosures about the study and an opt-out option were available for the patients on the hospital website. The study was conducted with full consideration of protecting patients’ personal information according to the privacy policy guidelines.

### Investigation of antimicrobial use history and susceptibility testing

A total of 58 *S. maltophilia* clinical isolates and the corresponding patients were enrolled in this study. We evaluated the antimicrobial susceptibility of *S. maltophilia* isolates, antimicrobial use history within 30 days prior to isolation of *S. maltophilia*. In this study, antimicrobials used for more than 3 days were counted as “use” cases. All bacterial species were identified using conventional methods and/or a MALDI Biotyper (Bruker Daltonics, Billerica, MA, USA). Antimicrobial susceptibility tests were conducted using MicroScan Neg MIC3J (Beckman Coulter, Inc., CA, USA) and Microscan WalkAway plus System (Beckman Coulter, Inc., CA, USA). The results were interpreted based on the Clinical and Laboratory Standards Institute guidelines M100-S30.

### Comparison of the *S. maltophilia* infection and colonization groups

*S. maltophilia* infection was defined as a positive microbiological culture from one or more collected sterile specimens. In cases with positive urine cultures, gram stain evidence of phagocytosis, fever, and abdominal symptoms were considered in the determination of infection or colonization. Cases in which *S. maltophilia* was detected in sputum were defined as infection (*S. maltophilia*-associated pneumonia) based on the criteria of previous studies [17]. Briefly, *S. maltophilia*-associated pneumonia was diagnosed via a positive microbiological culture accompanied by radiographic signs on chest X-ray or a computed tomography scan of pulmonary infection (presence of new or increasing infiltrates on chest radiograph) along with one or more of the following symptoms: fever (≥ 38 °C) or hypothermia (< 35 °C); new cough with or without sputum production, pleurisy chest pain, dyspnea, and altered breathing sounds on auscultation.

*S. maltophilia* colonization was defined as a positive microbiological culture for *S. maltophilia* without the above clinical signs of infection. In this study, the SM group included patients with *S. maltophilia* infection, and the colonization group included patients with *S. maltophilia* colonization. The following factors associated with *S. maltophilia* infection were obtained from clinical data: Sequential Organ Failure Assessment (SOFA) score at the time of *S. maltophilia* isolation; history of ICU admission, history of surgery, presence of tracheal intubation, presence of central venous (CV) catheter insertion within 30 days prior to *S. maltophilia* isolation; neutropenia (neutrophil count < 500/μL) within 14 days prior to *S. maltophilia* isolation; and length of hospital stay up to the time of *S. maltophilia* isolation were recorded. In addition, a history of chronic obstructive pulmonary disease, diabetes, cerebrovascular disease, cardiovascular disease, and chronic kidney disease was analyzed. History of drug administration within 30 days prior to *S. maltophilia* isolation, including steroid, chemical, antimicrobial, or immunosuppressive therapy, was also investigated. Mortality was defined as death within 7–30 days of *S. maltophilia* isolation. The results obtained were compared between the SM and colonization groups.

### Statistical analysis

Statistics analyses to determine the risk factors between the two groups were conducted using the χ^2^ test or Fisher's exact test for univariate comparisons and the Mann‒Whitney test for continuous variable comparisons. Survival rates were estimated using Kaplan‒Meier curves and compared between groups using the log-rank test. Statistical significance was set at *p* < 0.05. All analyses were performed using SPSS version 24 software (SPSS Inc., Chicago, IL, USA).

## Results

### Patient characteristics

A total of 58 patients with *S. maltophilia* isolates (median age, 80.5 years; age range, 49–100 years; 70.7% male) were enrolled in this study. The basic demographic data and characteristics of patients are listed in Table 1. Antimicrobial use in the 30 days prior to the isolation of *S. maltophilia* was observed in 55/58 (94.8%) patients, with 43.1, 31.0, 27.6, 25.9, 5.2, 3.4, and 0% of patients receiving meropenem, sulbactam/ampicillin, tazobactam/piperacillin, vancomycin, levofloxacin (LVFX), TMP-SMX, and minocycline (MINO), respectively.

**Table 1:** Demographic data and patients’ characteristics

Risk factor	Patients n (%)	*S. maltphilia* infection n (%)	*S. maltphilia* colonization n (%)	*p*-value
	(*n* = 58)	(*n* = 12)	(*n* = 46)	
Male, n	41 (70.7)	7 (58.3)	34 (73.9)	0.31
Age (median)	80.5	84	79	0.11
SOFA score (median)	3	5	3	0.01
ICU admission	15 (25.9)	2 (16.7)	13 (28.3)	0.71
Neutropenia (< 500/μL)	4 (6.9)	2 (16.7)	2 (4.3)	0.19
Days of hospitalization (median)	20.0	20.5	20.0	0.78
Surgery	19 (32.8)	3 (25.0)	16 (34.8)	0.73
Intratracheal intubation	9 (15.5)	2 (16.7)	7 (14.9)	1.00
Central venous catheterization	26 (44.8)	9 (75.0)	17 (37.0)	0.03
**Comorbidities**				
COPD	4 (6.9)	2 (16.7)	2 (4.3)	0.19
Diabetes	16 (27.6)	2 (16.7)	14 (30.4)	0.48
Cerebrovascular disease	17 (29.3)	0 (0.0)	17 (37.0)	0.01
Cardiovascular disease	27 (46.6)	5 (41.7)	22 (47.8)	0.76
Chronic kidney disease	10 (17.2)	4 (33.3)	6 (13.0)	0.19
**Previous drug exposure**				
Steroid	10 (17.2)	4 (33.3)	6 (13.0)	0.19
Anticancer agent	7 (12.1)	3 (25.0)	4 (8.7)	0.15
Fluoroquinolone	3 (5.2)	1 (8.3)	2 (4.3)	0.51
Carbapenem	28 (48.3)	7 (58.3)	21 (45.7)	0.53
Anti-MRSA drugs	17 (29.3)	3 (25.0)	14 (30.4)	1.00
Immunosuppression	1 (1.7)	0 (0.0)	1 (2.2)	1.00
**Mortality**				
7-day mortality	2 (3.4)	1 (8.3)	1 (2.2)	0.37
30-day mortality	8 (13.8)	5 (41.7)	3 (6.5)	0.01

*COPD* chronic obstructive pulmonary disease, *ICU* intensive care unit, *MRSA* methicillin-resistant *Staphylococcus aureus*, *SOFA* Sequential Organ Failure Assessment

### Bacterial strains detected along with *S. maltophilia*

In 40/58 patients (69.0%), strains other than *S. maltophilia* were simultaneously isolated; 27.7% were *Candida* spp. and 23.1% were *Corynebacterium* spp. (Table 2).

**Table 2:** Bacterial strains detected along with *Stenotrophomonas maltophilia*

Bacterial strain	Number of strains (%)	
	*n* = 65	
***Gram positive coccus***		
* Staphylococcus aureus*	6	(9.2)
* Staphylococcus epidermidis*	2	(3.1)
* Staphylococcus lugdunensis*	1	(1.5)
Coagulase-negative* Staphylococci*	1	(1.5)
* Enterococcus faecalis*	2	(3.1)
* Enterococcus faecium*	2	(3.1)
* Enterococcus raffinosus*	1	(1.5)
***Gram Positive Rod***		
* Corynebacterium spp.*	15	(23.1)
***Gram Negative Rod***		
* Pseudomonas aeruginosa*	3	(4.6)
* Klebsiella pneumoniae*	3	(4.6)
* Klebsiella aerogenes*	2	(3.1)
* Klebsiella oxytoca*	1	(1.5)
* Escherichia coli*	1	(1.5)
* Enterobacter cloacae*	2	(3.1)
Other *Enterobacter spp.*	1	(1.5)
* Citrobacter rodentium*	1	(1.5)
* Citrobacter werkmanii*	1	(1.5)
* Aeromonas eucrenophila*	1	(1.5)
* Providencia rettgeri*	1	(1.5)
***Fungus***		
* Candida albicans*	11	(16.9)
* Candida glabrata*	3	(4.6)
* Candida parapsilosis*	1	(1.5)
* Candida tropicalis*	1	(1.5)
Other *Candida spp.*	2	(3.1)

### Antimicrobial susceptibility of *S. maltophilia* isolates

Owing to missing data, only 42 of the 58 samples could be analyzed for antimicrobial susceptibility of ceftazidime (CAZ) and cefozopran (CZOP). The susceptibility rates of *S. maltophilia* were as follows: 28.6% to CAZ, 2.4% to CZOP, 100% to MINO, 96.6% to LVFX, and 98.3% to TMP-SMX (Table 3). There was no significant correlation between the history of antimicrobial use prior to *S. maltophilia* infection and antimicrobial resistance.

**Table 3:** Antimicrobial susceptibility of *Stenotrophomonas maltophilia*

	Sensitive Susceptible (%)	Intermediately Susceptible (%)	Resistant (%)
Ceftazidime (42 isolates)	12 (28.6)	5 (11.9)	25 (59.5)
Cefozopran (42 isolates)	1 (2.4)	0 (0.0)	41 (97.6)
Minocycline (58 isolates)	58 (100)	0 (0.0)	0 (0.0)
Levofloxacin (58 isolates)	56 (96.6)	1 (1.7)	1 (1.7)
Trimethoprim-Sulfamethoxazole (58 isolates)	57 (98.3)	0 (0.0)	1 (1.7)

### Case analysis of the *S. maltophilia* infection group

The SM group included 12/58 patients (20.7%), with 58.3% of the specimens from sputum, 25.0% blood, 8.3% pleural fluid, and 8.3% bile (Table 4). Invasive medical procedures were performed in 10/12 (83.3%) patients: 75.0% had CV catheter insertion, 25.0% underwent a surgical procedure, and 16.7% had tracheal intubation. Notably, all the patients from whom *S. maltophilia* was isolated from sterile sites had undergone CV insertion. Comorbidities included cerebrovascular disease (0.0%) and cardiac disease (41.7%). Concomitant medications included steroids (33.3%) and immunosuppressants (0.0%). In the SM group, all patients had a history of antimicrobial use prior to isolation of *S. maltophilia*. Eleven patients were treated, excluding one who was receiving end-of-life palliative care. The most commonly used antimicrobial agents included MINO (41.7%), LVFX (33.3%), and TMP-SMX (16.7%). Death within 30 days was observed in 4/5 (80.0%) of cases of *S. maltophilia* isolated from sterile sites in blood, pleural fluid or bile. Among the cases where *S. maltophilia* was detected in sputum, 1 out of 7 (14.3%) resulted in death within 30 days.

**Table 4:** Clinical features of patients with *Stenotrophomonas maltophilia* infection

Case no	Age	Sex	SOFA score	Culture	Susceptibility			History of patients within 30 days			Cerebrovascular disease	Cardiovascular disease	Drug use within 30 days			7 days dead	30 days dead	Treatment antibiotics
					LVFX	MINO	TMP-SMX	CV	Surgery	Intubation			Steroids	Immunosuppressive aget	Pre-adinimistration antibiotics			
1	94	F	5	blood	S	S	S	** + **	−	−	−	** + **	−	−	CTRX	−	−	MINO
2	92	M	1	sputum	S	S	S	−	** + **	−	−	−	−	−	SBT/ABPC, CTRX	−	−	LVFX
3	82	M	5	sputum	S	S	S	−	−	−	−	−	** + **	−	TAZ/PIPC, MEPM, LZD, VCM	−	−	MINO
4	70	F	9	sputum	S	S	S	** + **	−	−	−	−	−	−	CFPM, MEPM	−	−	LVFX
5	83	F	7	blood	S	S	S	** + **	−	−	−	** + **	−	−	SBT/ABPC	−	** + **	MINO
6	82	M	3	pleural effusion	S	S	S	** + **	** + **	** + **	−	−	** + **	−	TAZ/PIPC, MEPM, LVFX, TEIC, DAPT	−	** + **	TMP-SMX
7	92	M	7	sputum	S	S	S	** + **	−	−	−	** + **	−	−	CTRX, CLDM	** + **	** + **	MINO
8	84	M	4	sputum	S	S	S	** + **	−	−	−	** + **	−	−	DRPM	−	−	TMP-SMX
9	60	M	2	sputum	S	S	S	** + **	** + **	** + **	−	−	** + **	−	CTRX, CLDM, TAZ/PIPC, VCM	−	−	LVFX
10	84	F	0	blood	S	S	S	** + **	−	−	−	−	−	−	CMZ, DRPM	−	** + **	LVFX
11	95	F	5	sputum	S	S	S	−	−	−	−	** + **	** + **	−	MEPM	−	−	MINO
12	82	M	5	bile	S	S	S	** + **	−	−	−	−	−	−	SBT/CPZ, DRPM, DAPT	−	** + **	-

*CEZ* cefazolin, *CFPM* cefepime, *CLDM* clindamycin, *CMZ* cefmetazole, *CTRX* ceftriaxone, *CV* Central Vein, *DAPT* daptomycin, *DRPM* doripenem, *LVFX* levofloxacin, *LZD* linezolid, *MEPM* meropenem, *MINO* minocycline, *S* susceptible, *SBT/ABPC* sulbactam/ampicillin, *SBT/CPZ* sulbactam/cefoperazone, *SOFA* Sequential Organ Failure Assessment, *TAZ/PIPC* tazobactam/piperacillin, *TEIC* teicoplanin, *TMP-SMX* trimethoprim-sulfamethoxazole, *VCM *vancomycin

### Analysis of risk factors for *S. maltophilia* infection

**Fig. 1:**
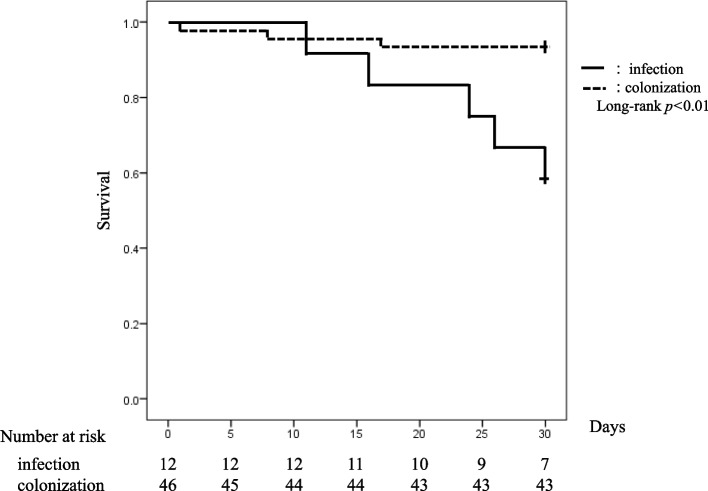
Kaplan–Meier survival curve for patients with *Stenotrophomonas maltophilia* infection (*solid line*) and colonization (*dotted line*); *p* < 0.01

Antimicrobial susceptibilities in the SM and colonization groups were as follows: CAZ, 25.0 and 29.4%; CZOP, 0 and 2.9%, MINO, 100 and 100%; LVFX, 100 and 95.7%; and TMP-SMX, 100 and 97.8%, respectively. Comparative data for the SM and colonization groups are shown in Table 1. SOFA score (median, 5; range, 0–9 vs. median, 3; range, 0–11; *p* = 0.01) and CV catheterization (75.0%, 37.0%; *p* = 0.03) were identified as risk factors for *S. maltophilia* infection. The SM group also had a significantly higher 30-day mortality rate than that of the colonization group (41.7%, 6.5%; *p* = 0.01). ANeutropenia within 14 days (16.7%, 4.3%; *p* = 0.19) was higher in the SM group than in the colonization group, but the difference was not significant. In contrast, cerebrovascular disease history was significantly higher in the *S. maltophilia* colonization group (0.0%, 37.0%; *p* = 0.01). Carbapenems were the most commonly used antimicrobial agents (58.3%, 45.7%). In contrast, fluoroquinolones were used by 8.3 and 4.3% of individuals in the SM and colonization groups, respectively. Non-antimicrobial concomitant medications included steroids (33.3%, 13.0%), antineoplastics (25.0%, 8.7%), and immunosuppressants (0%, 2.2%). Furthermore, the Kaplan‒Meier curve-estimated 30-day survival rate was significantly lower in the SM group (58.3%) than in the colonization group (93.5%) (*p* < 0.01; Fig. 1).

## Discussion

This study is one of the few reports investigating risk factors for *S. maltophilia* infection in acute care hospitals in Japan. High SOFA score and CV catheterization were risk factors for *S. maltophilia* infection. History of cerebrovascular disease was significantly higher in the *S. maltophilia* colonization group.

In our hospital, *S. maltophilia* was more frequently isolated from sputum. However, most of the cases were colonization, and infections were rare. Although there are other strategies to assess the severity of pneumonia, such as the Pneumonia Severity Index [18], CURB-65 [19], A-DROP [20], and I-ROAD [21], the SOFA score was used in this study because these scores overlap with certain assessment factors.

The present study identified elevated SOFA scores and the presence of CV catheters as risk factors for *S. maltophilia* infection. However, due to the limited number of cases of *S. maltophilia* infection, multivariate analysis of risk factors and outcomes was not feasible. The SOFA score and CV catheterization have been reported as risk factors for *S. maltophilia* infection in several previous studies [22–25].

In our study, a high rate of CV catheterization was observed in cases of *S. maltophilia* pneumonia, consistent with previous findings identifying CV catheterization as a risk factor for pneumonia in studies investigating *S. maltophilia* pneumonia among COVID-19 patients [26]. *S. maltophilia* produces biofilm and other virulence factors that enable colonization or infection in vulnerable hosts. The development of a biofilm is likely to be an important virulence factor associated with *S. maltophilia* infection [27, 28].

The results of this study suggest that *S. maltophilia* infection is strongly associated with disease severity and may be due to a combination of host and medical factors, including a history of antimicrobial use, high SOFA scores, and CV catheterization. Patients with *S. maltophilia* infection generally exhibit more severe symptoms compared to carriers and may play a role as a causative agent of opportunistic infections for other patients. Consequently, the implementation of enhanced monitoring procedures for patients exhibiting risk factors associated with *S. maltophilia* infection, in conjunction with the implementation of infection prevention measures, has the potential to result in a reduction of infection rates and the associated mortality rate. Previous studies have reported mortality rates of 12–49% for *S. maltophilia* infection [25, 29–31]. Consistent with previous literature, the mortality rate in our study was 36.4%. The 30-day mortality rate was found to be higher in cases where *S. maltophilia* was detected in the blood culture. The occurrence of fatalities was exclusively associated with cases involving CV catheterization. These findings underscore the necessity of meticulous monitoring of vascular access devices in patients with *S. maltophilia* detected from sterile sites and the implementation of an evaluation process to discern early indications of infection. In cases with high SOFA scores and CV catheterization, clinicians should give serious consideration to the possibility of *S. maltophilia* infection and initiate antimicrobial therapy even in cases where *S. maltophilia* is detected in sputum.

Antimicrobial susceptibility testing revealed that all isolates from the infected group demonstrated susceptibility to the antibiotics utilized in the treatment. Eleven patients were treated with MINO, LVFX, or TMP-SMX, with the exception of one patient who was terminally ill. Despite the administration of treatment, five patients died within 30 days. TMP-SMX is widely acknowledged as the preferred initial treatment for *S. maltophilia* infections. Despite the longstanding clinical experience with the use of TMP-SMX for *S. maltophilia* infections, several pharmacokinetic/pharmacodynamics (PK/PD) studies have emerged indicating that TMP-SMX is not bactericidal against *S. maltophilia*, even those with low TMP minimum inhibitory concentrations (MICs), regardless of the TMP-SMX dosage [32–34]. Similar limitations have been observed with other treatment options such as LVX and MINO [35–37]. Recent PK/PD studies call into question the current clinical breakpoints for TMP-SXT, LVFX, and MINO. In light of these findings, the 2024 guidance issued by the Infectious Diseases Society of America (IDSA) recommends the utilization of these agents exclusively as part of combination therapy. Studies have indicated that the combination of agents, such as cefiderocol, MINO, TMP-SMX, and fluoroquinolones, exhibits enhanced in vitro activity against *S. maltophilia* when compared to monotherapy [38]. In this study, the utilization of monotherapy may have contributed to unfavorable outcomes. However, given the high SOFA scores and the presence of CV catheterization, it is possible that the *S. maltophilia* infection was influenced by an unstable condition with a poor prognosis rather than a strong toxicity. Unfortunately, further analysis of the extant data was not possible.

The antimicrobial resistance rates of *S. maltophilia* in our hospital were 71.4% for CAZ, 3.4% for LVFX, 1.7% for TMP-SMX, and 0% for MINO. A previous study in Japan reported resistance rates of 67.4% for CAZ, 6.1% for LVFX, 17.7% for TMP-SMX, and 0% for MINO [39]; the TMP-SMX resistance rate was higher than that reported in our study, whereas other drug resistance rates were similar. A recent study has reported increasing resistance rates to LVFX, TMP-SMX, and MINO, and the emergence of strains resistant to two of these drugs [40]; therefore, future trends in antimicrobial resistance should be closely monitored. The mechanisms of resistance of *S. maltophilia* to fluoroquinolones involve multiple processes, including antimicrobial exposure [41], mutations in target sites of DNA gyrase and topoisomerase IV [42, 43], mechanisms such as drug efflux pumps [44, 45], and plasmid- or chromosome-mediated mutations in drug resistance genes [44–46]. In particular, among these drug-resistance genes, *Qnr* has the potential to transfer and confer quinolone resistance to other bacteria [47, 48].

According to data reported by the Japan Surveillance for Infection Prevention and Healthcare Epidemiology System (J-SIPHE) in Japan in 2023, the LVFX susceptibility rate for *S. maltophilia* was found to be 92.2% [49]. In the present study, the LVFX susceptibility rate was found to be 96.6%, a figure that similar with the data reported by J-SIPHE. The median AUD and DOT for fluoroquinolone use in 2023 were 2.1 and 2.4, respectively, whereas the AUD and DOT in our hospital were 7.0 and 7.9. This finding indicates that quinolone resistance in *S. maltophilia* may be influenced by factors other than antimicrobial exposure. In the present study, an investigation into resistance induced by antimicrobial exposure showed no significant differences in antimicrobial usage within 30 days prior to *S. maltophilia* isolation. A survey by the World Health Organization reported fluoroquinolone use (DDD/1000 inhabitation/day) in 2015 in Asia (Japan, 2.74; South Korea, 2.47), Europe (Italy, 3.8; France, 1.83), North America (Canada, 1.99), and Latin America (Brazil, 2.83) [50]. Farrell et al. investigated the susceptibility of *S. maltophilia* clinical isolates collected worldwide from 2003 to 2008 and reported that LVFX susceptibility rate was 78.0% in the Asia–Pacific region, 82.5% in North America, 83.7% in Europe, and 91.3% in Latin America [10]. Fluoroquinolone resistance does not necessarily correlate with usage or resistance rates. This finding suggests that factors other than antimicrobial exposure may be strongly involved and that the mechanism of acquiring resistance may differ among regions. Therefore, it is necessary to screen for fluoroquinolone resistance genes to obtain further data on antimicrobial resistance in *S. maltophilia*.

In addition, our investigation of bacteria isolated concurrently with *S. maltophilia* revealed that 31.0% were *Candida* spp. and 25.9% were *Corynebacterium* spp. Both species were isolated primarily in cases of compromised host defenses or microbial substitution due to the use of broad-spectrum antimicrobial agents. A few patients in this study had neutropenia. However, there was a high use of broad-spectrum antimicrobial agents, such as carbapenems and tazobactam/piperacillin, which may have contributed to the presence of *Candida* and *Corynebacterium* spp.

This study had several limitations. First, the sample size was small, and many risk factors were not statistically significant. Moreover, in severe cases where *S. maltophilia* was isolated, many strains were isolated simultaneously. However, the influence of concurrent strains on mortality risk remains unclear. Second, an accurate diagnosis of *S. maltophilia* pneumonia is difficult, and the definitions of *S. maltophilia* pneumonia vary among studies. Colonization may not have been completely excluded in this study. Finally, this was a single-center study, and the antimicrobial susceptibility of *S. maltophilia* was limited to strains detected in our hospital. In addition, no study was performed at the genetic level, and detailed data on antimicrobial resistance remain unclear. Regional differences in antimicrobial susceptibility have been reported, and further studies are warranted to evaluate the susceptibility of *S. maltophilia* in Japan.

## Conclusions

We investigated the risk factors for *S. maltophilia* infection and antimicrobial resistance in an acute care hospital in Japan. High SOFA score and CV catheterization were identified as risk factors for *S. maltophilia* infection. The 30-day mortality rate was significantly higher, and the survival rate was significantly lower in patients with *S. maltophilia* infection. The results of this study suggest that when *S. maltophilia* is isolated from severe cases or cases of CV insertion, early treatment with consideration of *S. maltophilia* infection is recommended. Therefore, a multicenter prospective cohort study is needed to identify the risk factors associated with mortality in *S. maltophilia* infection.

## Data Availability

The datasets generated and/or analyzed during the current study are available from the corresponding author upon reasonable request.

## Abbreviations

AUD: Antimicrobial use density
CAZ: Ceftazidime
CV: Central venous
CZOP: Cefozopran
DOT: Days of therapy
ICU: Intensive care unit
LVFX: Levofloxacin
MINO: Minocycline
SOFA: Sequential Organ Failure Assessment
TMP-SMX: Trimethoprim-sulfamethoxazole
